# Quality, Reliability, and Viewer Engagement of YouTube Videos on Esophageal Cancer: A Cross-Sectional Study

**DOI:** 10.1245/s10434-026-19996-1

**Published:** 2026-06-22

**Authors:** Sedat Carkit, Mustafa Karaagac

**Affiliations:** Department of General Surgery, Erciyes University Faculty of Medicine, Kayseri, Türkiye

**Keywords:** Esophageal cancer, YouTube, Online health information, Video content analysis

## Abstract

**Background:**

Esophageal cancer is associated with substantial morbidity and mortality worldwide and is frequently diagnosed at advanced stages, leading patients and their relatives to seek health-related information beyond traditional clinical encounters. In recent years, YouTube has become a popular source of medical information. Nevertheless, questions persist regarding the accuracy, credibility, and overall reliability of the content available on the platform.

**Methods:**

This cross-sectional study evaluated publicly available YouTube videos related to esophageal cancer. Data were collected on December 3, 2025, using a browser without a user login to minimize algorithm-driven bias. Viewer engagement metrics (views, likes, and comments), source categories, and country of origin were recorded for each video. Content quality and reliability were assessed using the DISCERN instrument, Journal of the American Medical Association (JAMA) benchmark criteria, and Global Quality Score (GQS). Non-parametric statistical analyses were used to compare quality outcomes across source categories and evaluate the correlations between the engagement metrics and quality scores.

**Results:**

A total of 78 videos met the inclusion criteria, most of which originated in the USA (83.3%). Health-related channels constituted the largest source category (35.9%), followed by patient experience-based videos (23.1%), and private institutions (20.5%). Viewer engagement metrics (views, likes, and comments) did not differ significantly among source types (p > 0.05). In contrast, the content quality varied substantially. Videos produced by public institutions achieved the highest DISCERN, JAMA, and GQS values, whereas patient-experience-based videos demonstrated significantly lower quality and reliability (p < 0.001). Engagement metrics were strongly intercorrelated but showed no association with quality scores.

**Conclusion:**

YouTube videos related to esophageal cancer frequently exhibit moderate informational quality, and popularity metrics do not reflect content reliability. Source credibility plays a critical role in determining video quality, underscoring the need for greater involvement of healthcare professionals and public institutions in digital health content production.

Esophageal cancer is a clinically challenging disease entity with a substantial global burden of morbidity and mortality, largely because of its frequent diagnosis at advanced stages and the limited effectiveness of available treatment options.^1^ The complexity of the diagnostic process, multidisciplinary nature of therapeutic approaches, and marked interindividual variability in prognosis contribute to an increased demand for disease-related information among patients and their relatives. In this context, individuals increasingly seek information not only through direct communication with healthcare professionals but also through digital platforms.^2^

The Internet and social media have become primary sources of rapid and easily accessible health information, particularly in recent years.^3^ Among video-based platforms, YouTube is one of the most widely utilized resources for patients, caregivers, and the general public because of its audiovisual format and extensive reach.^4^ However, the absence of formal scientific oversight of YouTube content raises significant concerns about the accuracy, reliability, and educational value of the information presented. This issue is especially critical in life-threatening conditions such as cancer, where incomplete or misleading information may adversely influence clinical decision-making processes and patient behavior.^5^

A growing body of literature has evaluated the quality, reliability, and educational value of medical content available on YouTube across various disease categories.^6^ Many of these studies have reported a lack of consistent association between popularity indicators and content quality and highlighted that the identity of the content producer, such as physicians, public institutions, private organizations, or patient-based sources, is a key determinant of informational quality.^7^ Nevertheless, studies providing a systematic and multidimensional evaluation of YouTube videos specifically focused on esophageal cancer remain limited.^8^

Online video content related to esophageal cancer commonly addresses critical topics, including disease symptoms, diagnostic methods, surgical and non-surgical treatment options, potential complications, and prognosis. Assessing such information in terms of scientific accuracy, timeliness, and objectivity is essential for both patients and health care professionals. Furthermore, clarifying how user engagement metrics such as views, likes, and comments relate to content quality may contribute to more effective guidance for digital health communication strategies.^9^

This study aimed to systematically evaluate YouTube videos related to esophageal cancer with respect to content quality, information reliability, and viewer engagement. Video content was analyzed using validated and widely applied assessment tools previously employed in the evaluation of medical information.^10^ Through this comprehensive approach, this study sought to provide insight into the current landscape of online health information on esophageal cancer and to offer guidance for the development of higher-quality digital health content in the future.

## Materials and Methods

This cross-sectional content analysis was conducted to examine the quality, reliability, and viewer engagement characteristics of YouTube videos addressing esophageal cancer. The analysis was limited to publicly accessible video materials and did not involve patient data or direct human participation. Therefore, ethical approval and informed consent were not required.^11^

Videos were retrieved and data collected on December 3, 2025. All video characteristics were recorded on the same day to ensure that the data reflected a single time point, given the continuously changing nature of the YouTube metrics. To minimize personalization effects, searches were conducted without logging into a user account or through a browser with a clear history and cookies. The videos were listed according to YouTube’s default relevance-based sorting method. All results obtained using the keyword “esophageal cancer” were screened, and those meeting the predefined eligibility criteria were included in the final analysis. The keyword “esophageal cancer” was selected because it represents the most commonly used and broadly recognized English-language search term for this disease among patients and the general public. The use of a single standardized keyword was intended to improve methodological reproducibility, minimize overlap between related search terms, and reduce the likelihood of duplicate video retrieval. Similar approaches using a primary disease-specific keyword have been adopted in previous YouTube-based studies evaluating online medical information quality.

For each eligible video, data were recorded using a structured collection form. The extracted variables include the number of views, likes, comments, time since upload (in months), country of origin, and uploader type. All engagement data were obtained directly from publicly available information on video pages. All recordings were completed within the same day to reduce variability related to temporal changes.

Videos were classified into five categories based on the identity of the uploader and the nature of the content: public institutions (e.g., universities, state hospitals, and official health organizations), private institutions (e.g., private hospitals and commercial healthcare providers), physicians (individual accounts clearly linked to licensed medical professionals), patient experience–based content (personal accounts shared by patients or relatives), and independent health-related channels without formal institutional affiliation. This classification was determined by reviewing the channel descriptions, video content, and presentation style.

**Fig. 1:**
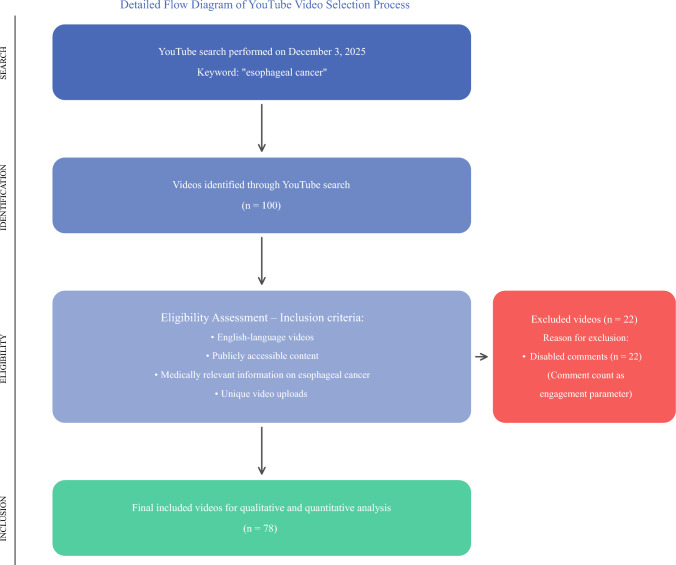
Detailed flow diagram illustrating the search strategy, eligibility assessment, exclusion process, and final inclusion of YouTube videos analyzed in the study

Videos were considered eligible for inclusion if they (1) primarily addressed esophageal cancer; (2) contained medically relevant information related to diagnosis, treatment, prognosis, or patient education; (3) were publicly accessible; (4) were presented in English; and (5) represented unique video uploads. Videos were excluded if they (1) were unrelated to esophageal cancer, (2) consisted primarily of promotional or advertising content, (3) lacked substantive clinical or educational information, (4) were duplicate uploads, or (5) had disabled comments, because comment count was included as a predefined viewer engagement parameter. Of the first 100 videos screened, 22 videos were excluded because of disabled comments, leaving 78 videos for the final analysis (Figure 1).

Content quality and reliability were assessed using the three established instruments. The DISCERN tool was used to evaluate the accuracy, balance, and overall informational value of the videos.^12^ The Journal of the American Medical Association (JAMA) benchmark criteria were used to assess the reliability in terms of authorship, source attribution, currency, and transparency.^13^ Overall educational value and presentation quality were measured using the Global Quality Score (GQS), which reflects informational completeness, clarity, and usefulness for viewers.^7^

Two reviewers with clinical and academic experience independently evaluated all the videos. Before the formal assessment, a calibration exercise was conducted using a randomly selected subset of videos to standardize scoring practices. Interobserver agreement was measured using Cohen’s kappa coefficient and interpreted according to established thresholds: <0.40 poor, 0.41–0.60 moderate, 0.61–0.80 good, and >0.81 excellent agreement.^14^

### Statistical Analysis

All statistical analyses were performed using IBM SPSS Statistics for Windows, version 27.0 (IBM Corp., Armonk, NY, USA). The normal distribution suitability of continuous data was evaluated using the Kolmogorov–Smirnov and Shapiro–Wilk tests. Numerical variables conforming to a normal distribution are expressed as mean ± standard deviation, whereas those deviating from normality are presented as median (min–max).

The Kruskal–Wallis test was used to assess statistically significant differences among the groups. When a significant overall difference was detected, appropriate post hoc analyses were performed to conduct pairwise comparisons and identify the specific groups contributing to the observed significance. Correlations between continuous variables were evaluated using Spearman’s rank correlation coefficient (Spearman’s rho). Interobserver agreement between reviewers was assessed using Cohen’s kappa coefficient. Statistical significance was set at p<0.05.

## Results

Most of the videos originated from the USA (n = 65 [83.3%]), followed by India (n = 5 [6.4%]), the UK (n = 4 [5.1%]), Canada (n = 3 [3.8%]), and the Philippines (n = 1 [1.3%]).

In terms of source type, health-related channels constituted the largest proportion of the videos (n = 28 [35.9%]). This was followed by patient-experience-based content (n = 18 [23.1%]) and private institutions (n = 16 [20.5%]). Physician-generated videos accounted for 12.8% of the sample (n = 10), whereas public institutions contributed the fewest (n = 6 [7.7%]).

The viewer engagement metrics demonstrated substantial variability. The mean number of views was 83,741.47 ± 318,210.00 (range 61–2,718,168). The videos received a mean of 1,091.92 ± 3,763.40 likes (range 2–26,000) and 92.23 ± 249.90 comments (range 1–1693). The mean time since upload was 63.23 ± 48.63 months (range 4–186).

The interobserver agreement analysis demonstrated good consistency between the two reviewers, with a Cohen’s kappa coefficient of 0.78.

Regarding content quality, the average DISCERN score was 45.72 ± 11.40 (range 28–67), the average JAMA score was 2.18 ± 0.99 (range 0.5–4.0), and the mean GQS was 3.22 ± 0.82 (range 1.5–4.5) (Table 1).

**Table 1:** Descriptive statistics of video characteristics

Variable	Mean ± SD	Minimum	Maximum
Views	83,741.47 ± 318,210.00	61	2,718,168
Likes	1091.92 ± 3763.40	2	26,000
Comments	92.23 ± 249.90	1	1693
Video age (months)	63.23 ± 48.63	4	186
DISCERN score	45.72 ± 11.40	28	67
JAMA score	2.18 ± 0.99	0.5	4.0
GQS	3.22 ± 0.82	1.5	4.5

Video age represents the time elapsed since the upload. DISCERN, the JAMA, and the GQS were used to assess the informational quality, reliability, and overall educational value of video content

GQS, Global Quality Score; JAMA, Journal of the American Medical Association; SD, standard deviation

No statistically significant differences were found between the source categories with respect to view count, such as count, comment count, or video age (p = 0.925, p = 0.910, p = 0.797, and p = 0.088, respectively). Nevertheless, videos produced by health-related channels tended to show higher mean views and counts, whereas those produced by public institutions and physicians generally exhibited lower engagement.

In contrast, differences were marked across the source categories for all the quality assessment instruments. Videos produced by public institutions achieved the highest DISCERN scores (65.0 ± 0.76), followed by private institutions (57.3 ± 0.26) and physician-generated content (54.7 ± 0.29). Patient experience–based videos demonstrated the lowest DISCERN scores (30.3 ± 0.33) (p < 0.001).

Distribution was also comparable for JAMA benchmark scores (p < 0.001). Videos produced by public institutions and physicians achieved the highest mean scores (3.67 ± 0.11 and 3.00 ± 0.17, respectively), whereas content based on patient experiences exhibited the lowest performance (0.86 ± 0.09).

**Fig. 2:**
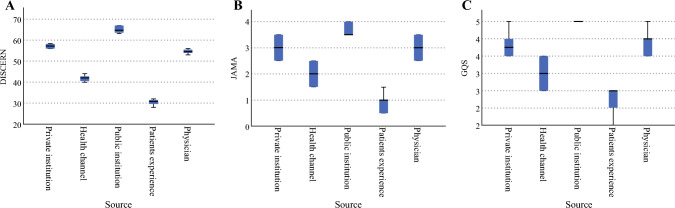
Distribution of (A) DISCERN and (B) Journal of the American Medical Association (JAMA) scores according to video source type. (C) Distribution of Global Quality Score (GQS) values across different video source categories

GQS differed significantly across video source categories (p < 0.001). Videos originating from public institutions demonstrated the highest overall quality (mean GQS 4.50 ± 0.00), whereas patient experience–based videos were associated with the poorest quality ratings (2.19 ± 0.10) (Table 2) (Figure 2).

**Table 2:** Comparison of video characteristics and quality scores according to source type

Variable	Private institution	Health channel	Public institution	Patient experience	Physician	p-Value
Views	74,691.8 ± 40,218.0	151,726.3 ± 97,197.6	29,067.0 ± 14,658.6	37,390.7 ± 15,304.5	31,439.9 ± 13,092.0	0.925
Likes	979.6 ± 643.2	1312.6 ± 929.5	205.7 ± 116.9	219.9 ± 63.8	2851.7 ± 1815.8	0.910
Comments	155.9 ± 112.2	102.3 ± 48.9	27.2 ± 11.9	66.7 ± 19.7	53.5 ± 25.3	0.797
Video age (months)	88.6 ± 15.7	70.9 ± 8.6	28.8 ± 10.4	50.9 ± 11.5	49.9 ± 8.7	0.088
DISCERN score	57.3 ± 0.26	41.8 ± 0.23	65.0 ± 0.76	30.3 ± 0.33	54.7 ± 0.29	<0.001
JAMA score	3.00 ± 0.12	1.96 ± 0.08	3.67 ± 0.11	0.86 ± 0.09	3.00 ± 0.17	<0.001
GQS	3.83 ± 0.09	3.04 ± 0.09	4.50 ± 0.00	2.19 ± 0.10	3.90 ± 0.10	<0.001

Values are presented as mean ± standard deviation. Comparisons among source types were performed using the Kruskal–Wallis test. Statistical significance was set at P<0.05. Video age represents the time elapsed since the upload. DISCERN, the JAMA, and the GQS were used to evaluate the informational quality, reliability, and overall educational value of the videos.

GQS, Global Quality Score; JAMA, Journal of the American Medical Association.

Correlation analysis revealed strong positive relationships among the engagement metrics. The view count was strongly correlated with both the like count (r = 0.878, p < 0.001) and the comment count (r = 0.858, p < 0.001). Likewise, likes and comments strongly correlated (r = 0.781, p < 0.001).

In contrast, no significant associations were identified between engagement measures (views, likes, and comments) and quality scores (DISCERN, JAMA, and GQS) (all p > 0.05). This finding indicates that higher audience interaction does not necessarily reflect greater medical accuracy or reliability.

However, the quality assessment instruments showed a strong correlation. DISCERN scores were strongly correlated with JAMA (r = 0.902, p < 0.001) and GQS (r = 0.934, p < 0.001), and JAMA scores were also strongly correlated with GQS (r = 0.809, p < 0.001). Together, these results confirm the consistency of the tools used to assess informational quality (Table 3).

**Table 3:** Spearman correlation analysis between variables

Variable	Views	Likes	Comments	DISCERN	JAMA	GQS
Views	1.000	0.878**	0.858**	0.000	−0.028	0.012
*p value*	—	<0.001	<0.001	0.998	0.809	0.915
Likes	0.878**	1.000	0.781**	0.061	−0.009	0.077
*p value*	<0.001	—	<0.001	0.596	0.935	0.504
Comments	0.858**	0.781**	1.000	−0.057	−0.103	−0.020
*p value*	<0.001	<0.001	—	0.620	0.372	0.865
DISCERN	0.000	0.061	−0.057	1.000	0.902**	0.934**
*p value*	0.998	0.596	0.620	—	<0.001	<0.001
JAMA	−0.028	−0.009	−0.103	0.902**	1.000	0.809**
*p value*	0.809	0.935	0.372	<0.001	—	<0.001
GQS	0.012	0.077	−0.020	0.934**	0.809**	1.000
*p value*	0.915	0.504	0.865	<0.001	<0.001	—

Values represent Spearman’s rank correlation coefficient (r). The corresponding p-values are shown below each correlation coefficient. All tests were two tailed

GQS, Global Quality Score; JAMA, Journal of the American Medical Association

^**^ p < 0.01

## Discussion

This study provides a comprehensive cross-sectional analysis of YouTube video content related to esophageal cancer, evaluating its quality, reliability, and viewer engagement in a multidimensional manner. The findings demonstrate a clear discrepancy between the popularity of online video content and its scientific quality and informational reliability while underscoring the decisive role of the content producer’s profile in determining overall quality.^15^

The predominance of videos originating from the USA was consistent with the dominant representation of English-language health-related content on YouTube and the high volume of content produced by US-based creators.^16^ Simultaneously, the presence of videos from several other countries highlights the platform’s international scope as a source of medical information. These findings demonstrate that YouTube serves as an internationally accessible source of esophageal cancer–related information reaching a broad and diverse audience.

Evaluation of video sources showed that the majority of the content was uploaded by health-related channels without direct institutional affiliation. This was followed by patient-experience-based videos and content produced by private healthcare institutions, indicating that a considerable portion of information is delivered outside formal academic or public health frameworks.

By contrast, videos uploaded by physicians and public institutions were relatively underrepresented. This distribution demonstrated that a substantial proportion of esophageal cancer–related information on YouTube originated from sources without direct professional or institutional affiliation.^17^ In the present study, patient experience–based videos constituted 23.1% of all included videos, representing one of the largest source categories. Despite this high representation, these videos demonstrated the lowest DISCERN, JAMA, and GQS scores among all source types. This discrepancy demonstrated that widely represented patient-centered content was associated with lower informational quality and reliability scores compared with institution- or physician-based videos.^18^

The evaluation of the quantitative engagement metrics showed wide variability in view counts, likes, and comments. The high standard deviation observed in the average view counts indicates that, although a small number of videos achieved exceptionally high visibility, many others received limited audience engagement. The absence of statistically significant differences in engagement metrics across source categories suggests that viewer behavior was not significantly associated with the credentials of the content producer. However, the relatively higher mean view and like counts observed for health channel–based videos was associated with higher mean engagement values among health channel–based videos.^19^

In contrast, marked and statistically significant differences were observed across source categories with respect to DISCERN, JAMA, and GQS scores, which reflect content quality and information reliability. Videos produced by public institutions and physicians consistently achieved the highest scores across all quality assessment tools, indicating that these sources tend to provide more balanced, transparent, and scientifically grounded information.^20^ Conversely, patient experience–based videos demonstrated the lowest quality scores across all instruments, indicating lower educational quality and reliability scores in patient experience–based content.^15^ The magnitude of the quality differences observed across source categories was particularly notable. Public institution videos achieved a mean DISCERN score of 65.0, compared with only 30.3 in patient experience–based videos. Similarly, the mean GQS values ranged from 4.50 in public institution videos to 2.19 in patient experience–based content. These findings indicate that source type was not only statistically associated with quality metrics but also demonstrated substantial practical differences in informational reliability and educational value.

These findings are largely consistent with those of prior studies that evaluated YouTube medical content across various disease categories.^21^ Similarly, previous research has reported that highly engaging videos do not necessarily convey higher quality or more reliable information, whereas content produced by academic or official sources tends to demonstrate superior informational quality.^22^ The present results extend this established pattern to esophageal cancer, indicating that this phenomenon also applies to relatively rare malignancies associated with high mortality. The present findings may be particularly relevant in the context of esophageal cancer because this disease is frequently associated with delayed diagnosis, complex multimodal treatment strategies, substantial nutritional challenges, and high mortality rates. Unlike several more common malignancies, patients with esophageal cancer often seek detailed information regarding dysphagia, surgical procedures, neoadjuvant therapy, postoperative recovery, and long-term prognosis. In this setting, exposure to incomplete or low-quality online information may have a greater impact on patient understanding and expectations. Therefore, the identification of substantial differences in informational quality across video source categories may carry specific clinical importance for esophageal cancer–related digital health communication.

Spearman’s correlation analysis revealed strong positive associations among engagement metrics, confirming that views, likes, and comments represent interrelated indicators of video popularity. However, the lack of significant correlations between these engagement measures and DISCERN, JAMA, or GQS scores clearly indicates that audience interest does not parallel informational quality.^23^ This finding demonstrated that higher viewer engagement did not correspond to higher informational quality or reliability scores.^24^

Notably, the strong correlations observed among quality assessment instruments merit attention. The robust positive associations between DISCERN, JAMA, and GQS scores indicate that, despite assessing distinct dimensions, these tools consistently reflect overall content quality.^25^ This observation supports the methodological validity and reliability of using these instruments in combination when evaluating YouTube-based medical information.^26^

This study had several important clinical and societal implications. In diseases such as esophageal cancer, which are characterized by poor prognosis and complex treatment pathways, previous studies have shown that patients and their relatives frequently seek online health information and digital educational resources.^2,3^ However, the present findings demonstrate that the most frequently viewed or engaged videos do not necessarily provide reliable or high-quality information.^4,27^ This raises concerns regarding the potential impact of inaccurate or incomplete information on patient expectations, treatment adherence, and clinical decision-making processes.^28^ In the present study, videos produced by physicians and public institutions demonstrated higher informational quality scores than patient experience–based content. These findings support the potential value of greater participation by professional and institutional sources in the development of evidence-based online health information. From a clinical perspective, these findings highlight the importance of proactively guiding patients toward reliable online information sources during routine oncological care. Patients diagnosed with esophageal cancer frequently seek supplementary information regarding symptoms, treatment options, prognosis, and postoperative expectations through digital media platforms. Exposure to inaccurate or low-quality content was associated in previous literature with misunderstanding of treatment strategies, unrealistic expectations, increased anxiety, and reduced adherence to evidence-based medical recommendations. Therefore, physicians should remain aware that online video content may influence health-related perceptions and expectations among viewers seeking medical information. In addition, healthcare institutions and professional organizations may play a more active role in developing accessible, scientifically accurate, and patient-oriented educational materials on widely used digital platforms such as YouTube.

This study had several limitations. First, owing to the dynamic nature of YouTube, the analysis reflects video characteristics and engagement metrics obtained at a single specific time point. Because YouTube content, ranking algorithms, and viewer interaction metrics continuously evolve, the visibility, popularity, and informational characteristics of videos may change over time. Therefore, the present findings should be interpreted as a cross-sectional snapshot of the platform during the study period rather than a permanent representation of available content.

Second, only English-language videos were included in the analysis. Although English constitutes one of the most widely used languages for online medical information, this selection may limit the generalizability of the findings to non-English-speaking populations and other regional digital health environments. The quality and reliability of esophageal cancer–related content may differ substantially across languages, cultures, and healthcare systems.

Finally, although the assessment tools used are widely accepted and valid for evaluating information quality and reliability, they do not directly capture individual viewers’ perceptions or subjective interpretations of content.

## Conclusion

This study demonstrated that a substantial proportion of YouTube videos related to esophageal cancer provide information of only moderate quality and reliability, whereas popularity indicators do not reflect content quality. Higher quality scores observed in videos produced by public institutions and physicians underscore the importance of information sources in delivering reliable health information. These findings offer valuable guidance for healthcare professionals and policymakers in shaping and directing digital health content and provide a foundation for future efforts aimed at improving the quality of online information in the field of esophageal cancer.
